# Performance Evaluation of Shotcrete Mortar with Silicon Manganese Slag as Substitute for Fine Aggregate

**DOI:** 10.3390/ma18081754

**Published:** 2025-04-11

**Authors:** Woo-Ri Kwon, Jung-Bin Lee, Bok-Mo Yoon, Jang-Ho Jay Kim

**Affiliations:** 1 Department of Civil and Environmental Engineering, Yonsei University, Seoul 03722, Republic of Korea; uskwon@yonsei.ac.kr (W.-R.K.); jbiiee@naver.com (J.-B.L.); 2 Miju Ecorock Co., Ltd., 36 Seohanam-ro 43beon-gil, Hanam 12991, Gyeonggi-do, Republic of Korea; miju@ecorock.co.kr

**Keywords:** aggregate replacement, silicon manganese slag, shotcrete, durability, workability, constructability, sustainability

## Abstract

Shotcrete is a versatile construction material, yet its performance limitations, such as high rebound rates and poor adhesion, demand technological improvements to ensure structural reliability. Silicon manganese (SiMn) slag, a by-product of SiMn alloy production, has gained attention as a potential sustainable alternative to natural aggregates in construction materials, addressing both resource depletion and carbon reduction challenges in the industry. This study is conducted to develop and evaluate a new mix design of mortar incorporating SiMn slag as fine aggregate, focusing on enhancing performance. Mixtures with varying percentages (0%, 30%, 50%, 70%, and 100%) of SiMn slag as a fine aggregate replacement were evaluated for fresh properties (air content, slump), mechanical performance (compressive strength, flexural strength, splitting tensile strength), durability (chloride ion penetration resistance, freeze–thaw resistance, carbonation resistance), and constructability (rebound rate, free shrinkage) to assess suitability as mortar for shotcrete. The experimental results demonstrated that the mixture with 50% SiMn slag replacement demonstrated the most balanced performance, showing an increase of 12.33% in compressive strength, 8.97% in splitting tensile strength, and 18.4% in flexural strength compared to the control. Durability properties also improved by an average of 11.93%, while rebound rate and shrinkage were significantly reduced. The findings confirm that SiMn slag is a technically viable and advantageous substitute for fine aggregates in shotcrete. Further research is needed to refine its economic feasibility and broaden its implementation in sustainable construction.

## 1. Introduction

The worsening climate crisis and global resource depletion have placed growing pressure on the construction industry to adopt sustainable and durable materials. As Pierre Humbert of the University of Oxford stated, “Let’s get this on the table right away, without mincing words. With regard to the climate crisis, yes, it’s time to panic” [1]. This urgent message highlights the responsibility of material engineers and researchers to contribute to low-carbon, resource-efficient construction practices. In this context, improving both the durability and sustainability of construction materials such as shotcrete is no longer optional but essential.

Shotcrete is broadly adopted in many different construction works, such as underground mining, tunnel lining, slope stabilization, and concrete repair strengthening due to its implementation flexibility, rapid application, and excellent bonding performance [2]. In the tunnel industry, shotcrete is primarily employed to form thin and flexible self-supporting layers inside tunnels [3] or as a tunnel lining serving as permanent support without the need for additional concrete members [4,5]. This application significantly enhances the safety of underground construction [6,7] while ensuring that projects are executed without unnecessary delays [8]. In addition, shotcrete offers significant advantages in performance and cost-effectiveness, particularly when applied to relatively light-load-bearing structures, such as landscaping tunnels, artificial rock formations, underground parking lots, and small-scale underground storage facilities. Shotcrete can achieve rapid construction as it does not require formwork, demonstrate adaptability to various structural design criteria, and result in reduced overall costs due to its minimal equipment needs. Despite its advantages, shotcrete encounters significant obstacles, such as poor adhesion [9], high rebound rate [10], and interface generation [11]. In 2022, a worker died at a railway construction site in Goyang, South Korea, due to shotcrete detachment. Also, a construction accident occurred in 2023, where a shotcrete wall collapsed at a condominium construction site in Coquitlam, Canada. Fortunately, no casualties were reported, but such incidents show the necessity of improving the performance of shotcrete to prevent recurring accidents.

**Figure 1:**
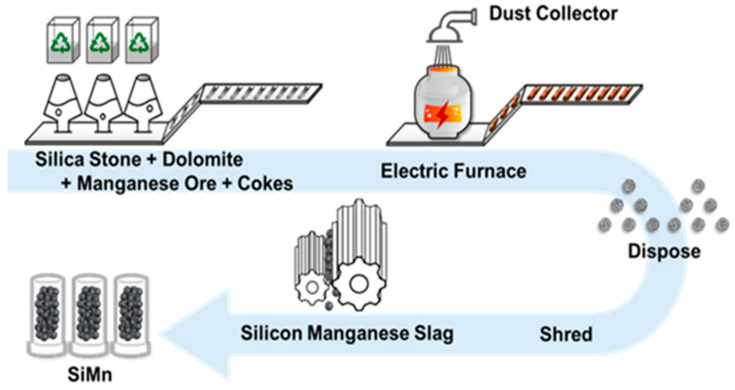
Production of silicon manganese (SiMn) slag.

The modern construction industry faces challenges, such as natural resource depletion and strict environmental regulations. For example, net-zero carbon emission goals emphasize the importance of sustainable construction [12]. In particular, the depletion of natural aggregates has driven the development of alternative aggregates through recycling waste resources, including disused aggregates, agricultural residues, and industrial wastes [13,14]. Recycling industrial waste, which places a significant burden on waste management systems due to the globally increasing volume of waste, not only reduces environmental pollution but also offers a sustainable solution by converting waste into valuable resources, thereby contributing to the transition toward a circular economy [15]. SiMn slag is a byproduct of silicon manganese production, which is generated in high-carbon ferro-manganese blast furnaces using manganese ore, silica stone, and coke [16]. SiMn slag is a waste resource frequently investigated for its potential reuse as aggregates in construction materials, along with blast furnace slag, steel slag, and fly ash [17]. The production process of SiMn slag is shown in Figure 1. The process involves producing silicon manganese in an electric furnace [18]. The remaining slag is cooled using high-pressure water and crushed for potential use [19]. As the global market for SiMn alloy production expands, the accumulation of SiMn slag has also increased, which poses environmental challenges due to its large volume [20,21,22]. Recycling SiMn slag provides solutions to the environmental and economic challenges caused by the production of silicon manganese, namely the need for large-scale treatment plants for the disposal of slag and generated fugitive dust and leachate [23]. To address this problem, many efforts have been made to recycle SiMn slag as a means of promoting resource circulation.

Several studies have positively evaluated the potential of using SiMn slag as a sustainable next-generation aggregate substitute. Wong et al. highlighted the potential for recycling SiMn slag as construction material [24]. Patil et al. further expanded its application, noting that SiMn slag is suitable for road pavement and railway tracks due to its high resistance to wear, abrasion, and degradation [25]. Similarly, Choi et al. demonstrated that mortar produced using rapid-cooled SiMn slag aggregate exhibits proper engineering properties, confirming its viability as a lightweight aggregate for construction [19]. Furthermore, Nath et al. suggested that SiMn slag can be used as both a binder and an aggregate in construction to enhance concrete properties [26].

The potential of SiMn slag as a sustainable substitute for fine aggregates in mortar for shotcrete was investigated to evaluate its performance. A new mix proportion incorporating SiMn slag as fine aggregates was developed through experimental research. Mix proportions were designed with varying SiMn slag replacement levels (0%, 30%, 50%, 70%, and 100%), and each mixture was evaluated to determine the most effective and practical formulation for shotcrete application. Fresh properties of the mortar were assessed through air content and slump tests. Mechanical properties of the mixtures were assessed through compressive strength, flexural strength, and splitting tensile strength tests, while durability was evaluated using chloride ion penetration resistance, freeze–thaw resistance, and carbonation resistance tests. Additionally, workability was examined through rebound rate and drying shrinkage tests to further understand the practical application. These evaluations identified the optimal mix proportion, ensuring superior performance and efficiency for practical shotcrete applications. The findings are expected to contribute to the development of high-performance, eco-friendly construction materials and provide a novel solution for sustainable construction and recycling waste.

## 2. Materials and Methods

### 2.1. Materials

A hazardous substance test conducted in accordance with waste process standards (No. 2015-50 from the Korean Ministry of Environment) indicates that SiMn slag is a harmless and eco-friendly material. As shown in Table 1, the only items detected in significant amounts were Pb, Cu, and Cd, and all 11 test items were below threshold values. The result of the test supports the suitability of SiMn slag as a harmless and eco-friendly substitute for fine aggregate.

The physical property test was carried out according to KS F 2504 (Standard Test Method for Density and Water Absorption Rate of Fine Aggregates) [27]. Table 2 shows that SiMn slag has a saturated surface-dry density of 3.00 g/cm^3^, an absolute dry density of 2.97 g/cm^3^, and a water absorption rate of 0.79%. The particle size of the SiMn slag used in this study ranged from 0.2 to 0.6 mm, which is included in the range defined for natural sand in KS F 2527 (Aggregates for Concrete) [28]. Compared to general fine aggregate, which has a specific gravity of 2.65 and a water absorption of 1%, SiMn slag exhibits higher specific gravity and a lower water absorption rate, indicating greater strength and enhanced resistance to freezing and thawing. This aspect suggests that SiMn slag has potential as a fine aggregate replacement for concrete and cement mortar.

To examine the chemical composition of SiMn slag, X-ray fluorescence spectrometry (XRF) was performed. XRF uses X-rays to emit electrons and identifies the atoms by filling the vacated electron spots with other electrons from X-rays to measure the energy of each atom’s unique value. In Table 3, a comparison of the chemical composition of SiMn slag and fine aggregate is shown. The results from the XRF analysis showed that SiO_2_, CaO, MnO, and Al_2_O_3_ are dominant elements in SiMn slag, with low levels of MgO, Fe_2_O_3_, and NaCl. However, fine aggregate is dominated by 77.2% of SiO_2_ and 10.2% of Al_2_O_3_, with low levels of CaO, MnO, MgO, Fe_2_O_3_, and NaCl. Therefore, SiMn slag can be defined as a latent hydraulic material, preventing alkaline aggregate reaction and reducing concrete expansion to minimize cracking. Overall, the findings suggest that SiMn slag has significant potential as an environmentally friendly and effective alternative to traditional fine aggregate in concrete and cement mortar.

### 2.2. Preliminary Test

Preliminary tests were conducted to determine the water–cement ratio for the mix and the required amount of plasticizer. In the initial assessment of the water–cement ratio for the mix, the results of the compressive strength test for various ratios were examined, as shown in Table 4. A mix proportion was formulated by adjusting the water–cement ratio within the range of 30% to 50% and focusing on the complete replacement of aggregate with SiMn slag. Compressive strength tests were conducted according to KS F 2405 (Test method for compressive strength of concrete) [29] on specimens cured for 3, 7, 14, and 28 days. The test results show that the highest compressive strength of 36.8 MPa at 28 days of age was obtained at the water–cement ratio of 35%. Also, a water–cement ratio of 40% showed a similar compressive strength of 35.8 MPa at 28 days of age.

The preliminary test to determine the required plasticizer amount for sufficient workability was carried out. The target slump value ranged between 12 mm and 15 mm. The tests were conducted with a plasticizer amount ranging from 0.3% to 0.9% of the cement weight, with 100% replacement of fine aggregate with SiMn slag. The slump test results are shown in Table 5. The results showed that the slump value of 13 mm, which corresponds to 0.5% plasticizer, meets the target slump value range. Thus, 0.5% plasticizer of the cement weight was selected as a final amount for the mix proportion.

### 2.3. Mix Proportion

To assess the performance of shotcrete with SiMn slag as the fine aggregate, preliminary compressive strength tests were conducted to determine a mix design. The mix designs listed in Table 6 are proposed to obtain a target compressive strength exceeding 21 MPa, with a slump range of 12–15 mm. Five cases with varying percentages of SiMn slag (0%, 30%, 50%, 70%, and 100%) as fine aggregate were considered. The water–cement ratio was set to 40%, and a plasticizer amount of 0.50% of the cement weight was determined from the previous preliminary tests. Ordinary Portland Cement (KS L 5201 Type 1) [30] and a polycarboxylate-based superplasticizer with air-entraining properties were used in this study. A reference specimen with 0% SiMn slag served as the control specimen (S-0), with the other specimens labeled as S-30, S-50, S-70, and S-100 based on the respective slag replacement percentages.

### 2.4. Test Methods

Air content and slump of the fresh mortar were evaluated for 0%, 30%, 50%, 70%, and 100% replacement of SiMn slag as the fine aggregate.

#### 2.4.1. Air Content Test

**Figure 2:**
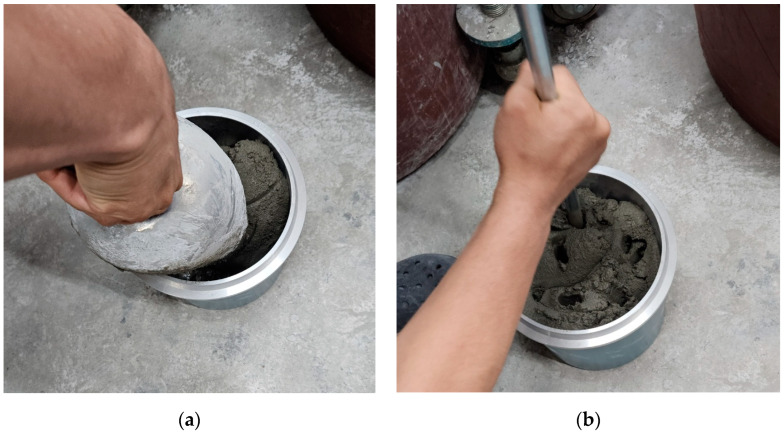
Air content test: (**a**) pouring mortar mix; (**b**) pounding mortar mix.

To determine the air content of the mortar, the air content measurement test was conducted following the guidelines of KS F 2421 (Standard Test Method for Air Content of Fresh Concrete by the Pressure Method) [31]. Figure 2 illustrates the air content test setup, including an air content meter and a cylindrical metal container with a minimum volume of 5 L. The air content of the mortar was calculated using Equation (1):

*A* = *A*_1_ − *G* (1)

where *A* (%) is the air content of fresh concrete; *A*_1_ (%) is the apparent content; *G* is the aggregate correction factor.

#### 2.4.2. Slump Test

**Figure 3:**
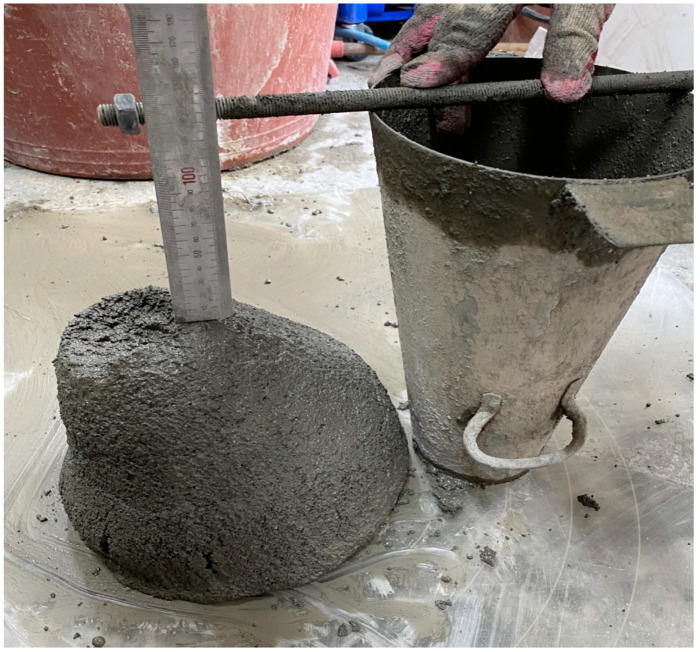
Slump test based on different plasticizer content.

The slump test for the mortar was conducted following the guidelines outlined in KS F 2402 (Test Method for Concrete Slump) [32]. The slump of the concrete was measured by the difference in height between the original height of the cone and the highest point of the slumped concrete, as shown in Figure 3.

To evaluate the mechanical properties of the hardened mortar, compressive, flexural, and splitting tensile strength tests were conducted.

#### 2.4.3. Compressive Strength Test

The compressive strength test for the mortar was conducted following the guidelines of KS F 2405. Cylindrical specimens were prepared according to the specifications outlined in KS F 2403 (Standard Test Method for Making Concrete Specimens) [33]. Mixes of the specimens contained various fine aggregate replacement ratios of 0%, 30%, 50%, 70%, and 100%. Specimens were wet-cured at a temperature of (20 ± 2) °C until the strength test. Before the compressive strength test, the top and bottom surfaces of the specimens were ground to be perpendicular to the compressive loading. A compressive strength test was performed using a Universal Testing Machine (UTM) with a maximum capacity of 1000 kN and precision of 500 N, under a constant loading rate of (0.6 ± 0.2) MPa/s. The compressive strength tests were conducted at 3, 7, 14, and 28 days of curing age. The compressive strength was calculated using Equation (2):

(2) $$f_{c}=P/\pi {\left(d/2\right)}^{2}$$

where *f_c_* (MPa) is the compressive strength of concrete; *P* (N) is the load; *d* (mm) is the diameter of the specimen.

#### 2.4.4. Flexural Strength Test

To assess the flexural strength of the mortar with SiMn slag as fine aggregate, the test was carried out following the guidelines of KS F 2408 (Standard Test Method for Flexural Strength of Concrete) [34]. Rectangular specimens with dimensions of 100 × 100 × 400 mm were prepared according to KS F 2403. The specimens were made with various aggregate replacement ratios of 0%, 30%, 50%, 70%, and 100%. The 3-point loading was used for the flexural strength test. The loading rate was maintained at (0.06 ± 0.04) MPa/s. Specimens were wet-cured at a temperature of (20 ± 2) °C until testing. Testing was conducted at 14 and 28 days of age, and flexural strength values were determined using Equation (3):

(3) $$f_{b}=PL/bh^{2}$$

where *f_b_* (MPa) is the flexural strength of concrete; *P* (N) is the maximum load from the testing machine; *L* (mm) is the span; *b* (mm) is the width of the specimen; *h* (mm) is the height of the specimen.

#### 2.4.5. Splitting Tensile Strength Test

The splitting tensile strength test was conducted following the guidelines of KS F 2423 (Standard Test Method for Splitting Tensile Strength of Concrete) [35]. Cylindrical specimens with a 100 mm diameter and 200 mm length were prepared in accordance with KS F 2403. Specimens were prepared with various aggregate replacement ratios of 0%, 30%, 50%, 70%, and 100%. Specimens were wet-cured at a temperature of (20 ± 2) ℃ until testing. A load with a rate of (0.06 ± 0.04) MPa/s was applied until the maximum load was reached. Splitting tensile strength tests were conducted at 14 and 28 days of age. The splitting tensile strength was calculated according to Equation (4):

(4) $$f_{sp}=2P/\pi dl$$

where *f_sp_* (MPa) is the splitting tensile strength of concrete; *P* (N) is the maximum load, as indicated by the testing machine; *Ɩ* (mm) is the span; *d* (mm) is the diameter of the specimen.

For durability, chloride ion penetration resistance, freezing, and thawing as well as accelerated carbonation tests were conducted.

#### 2.4.6. Chloride Ion Penetration Resistance Test

To assess the chloride ion penetration resistance of the mortar, the test was conducted following the guidelines of KS F 2711 (Standard Test Method for Resistance of Concrete to Chloride Ion Penetration by Electrical Conductance) [36]. Three cylindrical specimens were prepared for each SiMn slag replacement ratio, which were cured for 28 days. Table 7 outlines the evaluation standards for the total charge passage specified by KS F 2711. The total charge passage value was calculated using Equation (5):

(5) $$Q=900(I_{0}+2I_{30}+2I_{60}+\cdots +2I_{330}+I_{360})$$

where *Q* (C) is the charge passed in coulombs; *I*_0_ (A) is the current immediately after the voltage is applied; *I_t_* (A) is the current at t minutes after the voltage is applied.

#### 2.4.7. Freezing and Thawing Test

To evaluate the freezing and thawing resistance of the mortar with aggregate replacement, the test was conducted following the guidelines of KS F 2456 (Test Method for Resistance of Concrete to Rapid Freezing and Thawing) [37]. Freezing and thawing resistance was quantified by calculating the relative dynamic modulus using Equation (6):

(6) $$P_{c}=\left(n_{c}^{2}/n_{0}^{2}\right)\times 100$$

where *P_c_* (%) is the relative dynamic modulus of elasticity after freeze–thaw cycle *C*; *n_c_* (Hz) is the primary resonance frequency of the strain vibration after freeze–thaw cycle *C*; *n_0_* (Hz) is the primary resonance of strain vibration in freeze–thaw cycle 0.

#### 2.4.8. Accelerated Carbonation Resistance Test

**Figure 4:**
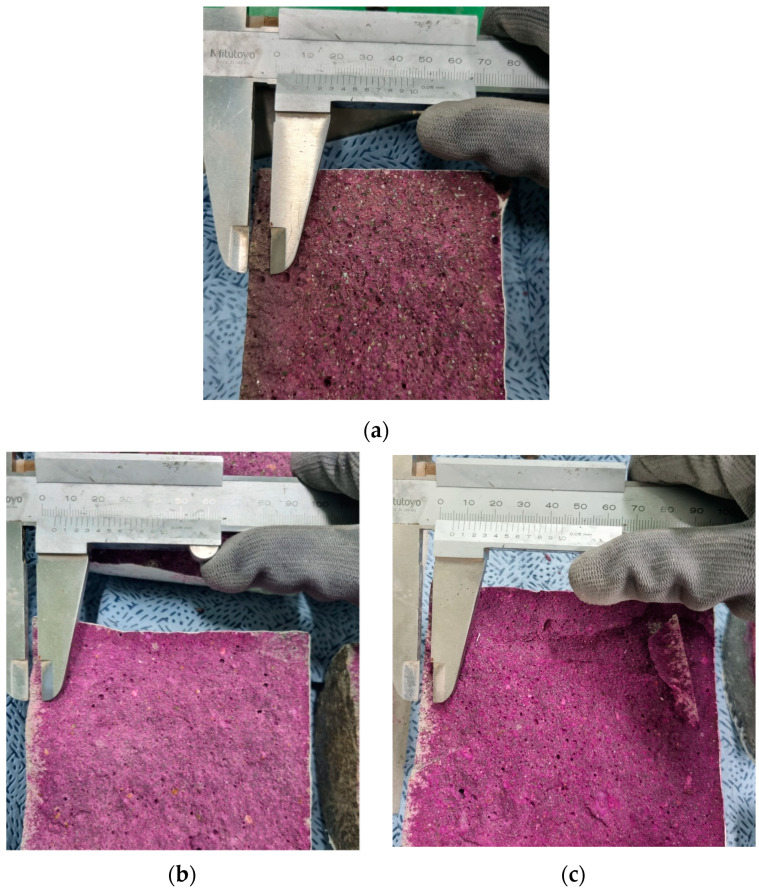
Carbonation depth measurement: (**a**) S-0 (control); (**b**) S-50; (**c**) S-100.

To assess the carbonation of the mortar, the test was conducted following KS F 2584 (Standard Test Method for Accelerated Carbonation of concrete) [38], as shown in Figure 4. Carbonization depth was measured after 4 weeks of exposure to carbon dioxide-filled test equipment by spraying phenolphthalein solution as a reagent. While typical times for carbonization depth measurement are 1, 4, 8, 13, and 26 weeks, the measurements were taken at a 4-week incubation period to correspond to the curing duration.

To evaluate the constructability of the mix proportion, rebound and drying shrinkage crack tests were conducted.

#### 2.4.9. Rebound Test

The rebound test was conducted to evaluate workability of the mortar. The mortar was shotcreted over a steel system consisting of steel bars and steel mesh, arranged in a grid pattern of 250 × 250 mm. The mortar was applied to the system over an area covering four grids with a thickness of 30 mm. To minimize the variability caused by operator skill, all spraying work was performed by a single skilled worker. The rebound amount was measured by collecting and weighing the mixture that fell to the ground after shotcreting was completed. The mixtures with water–cement ratios of 35% and 40% were tested, and rebound percentages were calculated using Equation (7):

(7) $$Rebound\;rate\;\left(\%\right)=\frac{Weight\;of\;rebound\;mixture}{Total\;weight\;of\;mixture}\times 100$$

#### 2.4.10. Drying Shrinkage Crack Test

**Figure 5:**
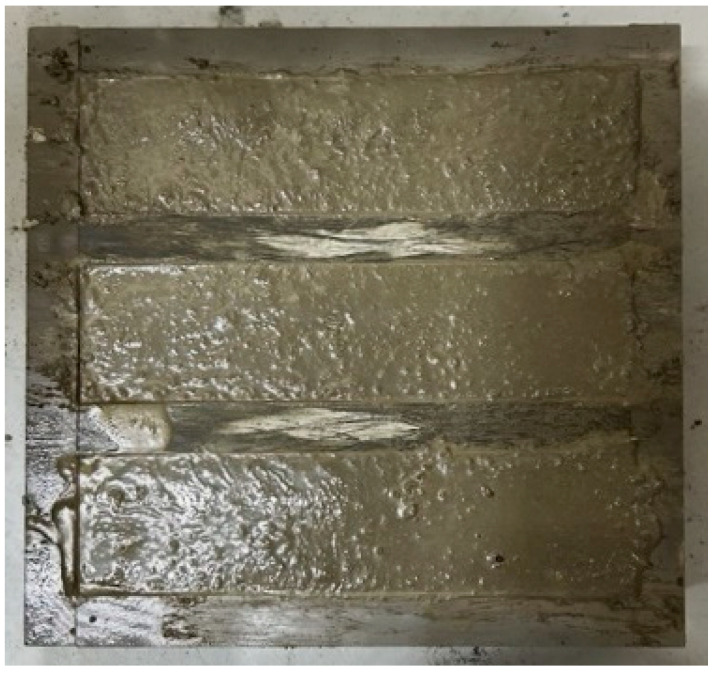
Free drying shrinkage crack test.

To evaluate the initial drying shrinkage performance of the mortar and to assess the initial shrinkage crack occurrence, the free shrinkage strain measurement test was conducted in accordance with KS F 2595 (Standard Test Method for Drying Shrinkage Crack in Concrete) [39]. As shown in Figure 5, the specimens were prepared with dimensions of 40 × 40 × 160 mm, which were attached with strain gauges to monitor strain over 30 days in a controlled environment of 23 °C temperature and 50% relative humidity.

## 3. Results

### 3.1. Fresh Properties

**Figure 6:**
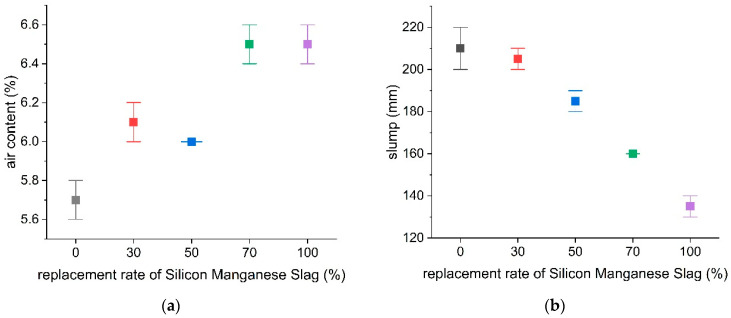
Air content and slump based on replacement rate of SiMn slag: (**a**) air content test; (**b**) slump test.

The air content and slump test results were tabulated and are given in Table 8. The air content and slump test results for all the specimens ranged between 5.7–6.6% and 130–220 mm, respectively, as shown in Figure 6. The control specimens without slag replacement exhibited the lowest air content average of 5.7%. As the SiMn slag replacement ratio increased incrementally from 30% to 100%, the air content correspondingly increased. More specifically, the S-30, S-50, S-70, and S-100 slag specimens had air contents of 6.1%, 6.0%, 6.3%, and 6.5% respectively. The full slag replacement S-100 specimens had the highest air content. All of the specimens displayed slump values exceeding 120 mm. The control specimens without slag substitution had the highest average slump of 210 mm. The S-30 specimens with a 30% fine aggregate replacement ratio exhibited a slightly lower but still high slump of 205 mm. Slump values progressively declined as higher SiMn slag contents were substituted. More specifically, the S-50, S-70, and S-100 specimens demonstrated slumps of 185 mm, 160 mm, and 135 mm, respectively. Therefore, an inverse correlation was observed between the slag replacement ratio and slump measurement, with the S-100 mixture having the lowest workability. Overall, greater SiMn slag substitution correlated with higher air content values and lower flowability and slump.

### 3.2. Mechanical Properties

**Figure 7:**
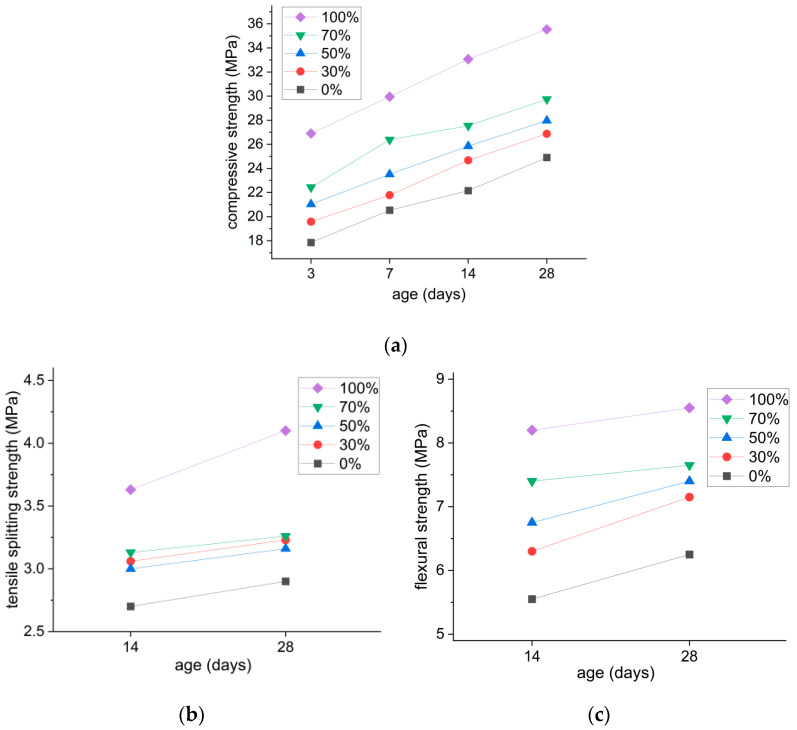
Compressive, splitting tensile, and flexural strength based on replacement rate of SiMn slag: (**a**) compressive strength; (**b**) splitting tensile strength; (**c**) flexural strength.

The compressive and splitting tensile strength test results are summarized in Table 9. Also, the flexural strength test results are summarized in Table 10. In all of the strength test results, the strengths increased as SiMn slag replacement ratios increased. As shown in Table 9, the control specimens without SiMn slag exceeded the minimum specified 21 MPa compressive strength at 28 days, averaging 24.9 MPa. Figure 7 illustrates superior strength development in the specimens with higher SiMn slag replacement ratios for all curing ages (3–28 days). In Figure 7a, the S-30, S-50, S-70, and S-100 specimens displayed 8%, 12%, 19%, and 43% higher 28-day compressive strengths than the strength of the control specimen, respectively. With respect to the splitting tensile strength results shown in Figure 7b, the same trend as that of the compressive strength results is demonstrated, with a 41.3% increase in the S-100 specimen compared to the control specimen. Likewise, the flexural strength results showed a similar trend with increased slag content, with S-100 specimens having the highest strength. At 28 days, the mortar with 30%, 50%, 70%, and 100% replacement ratios exhibited a 14%, 18%, 22%, and 37% flexural strength increase compared to the control specimen strength, respectively, as shown in Table 10 and Figure 7c. Thus, all of the investigated mechanical strengths were enhanced with increasing SiMn slag contents, verifying its feasibility and benefits as a shotcrete constituent for improved material performance.

### 3.3. Durability Properties

**Figure 8:**
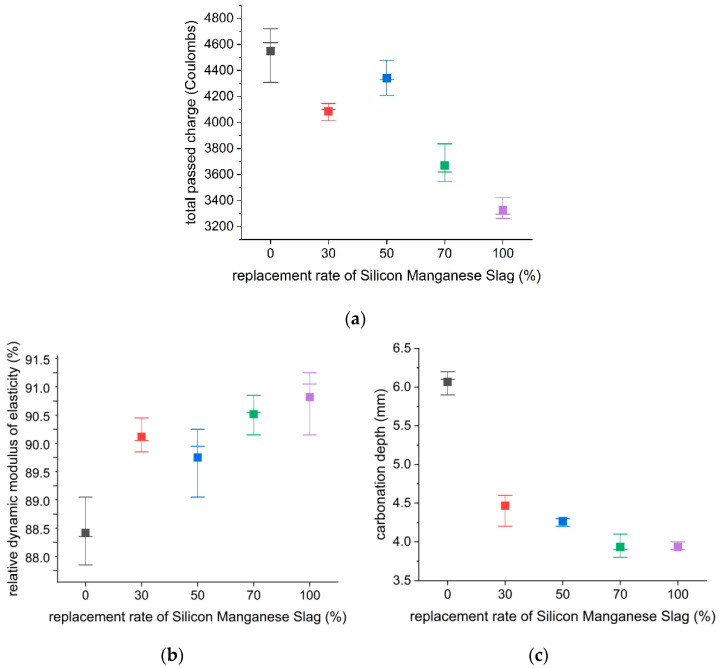
Chloride ion penetration resistance, freezing and thawing, and accelerated carbonation test based on replacement rate of SiMn slag: (**a**) chloride ion penetration resistance test; (**b**) freezing and thawing test; (**c**) accelerated carbonation test.

Durability performance of the mortar was evaluated under simulated environmental conditions. The durability tests of chloride ion penetration, freeze–thaw resistance, and accelerated carbonation were conducted. The results are tabulated in Table 11 and shown in Figure 8. In the accelerated chloride ion penetration test, the control specimen (0% SiMn slag) was applied with an average charge of 4546 coulombs for 28 days. As demonstrated in Figure 8a, the S-30, S-50, S-70, and S-100 specimens showed a 10%, 4%, 19%, and 27% decrease in chloride ion penetration, respectively, compared to the control specimen. The resulting trends showed that the chloride ion penetration resistance improved as the slag replacement ratio increased.

For freezing–thawing resistance evaluation of the mortar, the relative dynamic modulus of elasticity (RDME) was measured after 300 freeze–thaw cycles. In Table 11 and Figure 8b, the RDME results are shown as a percentage of the initial RDME measured from the specimens without a freeze–thaw cycle (0 cycle). The results showed that all of the measured RDMEs from the specimens after 300 cycles exceeded 90%, except the control specimen (0% SiMn slag). The increasing RDME results coincide with the SiMn slag content increase, with the largest average RDME of 91.07% obtained from the S-100 specimen. The results indicate that SiMn slag mortar has better freeze–thaw resistance than ordinary silica sand mortar when used in the shotcrete mortar mix.

For carbonation resistance evaluation, the specimens are exposed to a carbon dioxide environment using the accelerated carbonation test machine to measure carbonation depth. The carbonation depth results are tabulated in Table 11 and shown in Figure 8c. The results showed that the carbonation depth drastically decreased as the SiMn replacement ratio increased. The mortar with the replacement ratios of 0, 30, 50, 70, and 100% had average carbonation depths of 6.06, 4.45, 4.26, 3.93, and 3.86 mm, respectively. Even with 30% SiMn slag replacement of silica sand, an approximate 36.18% reduction of carbonation depth was achieved, showing a dramatic enhancement of carbonation resistance when using SiMn slag over silica sand. Thus, replacing fine aggregate with SiMn slag enhanced the resistance of the mortar to chloride intrusion, freezing damage, and carbonation in harsh exposures.

### 3.4. Constructability

**Figure 9:**
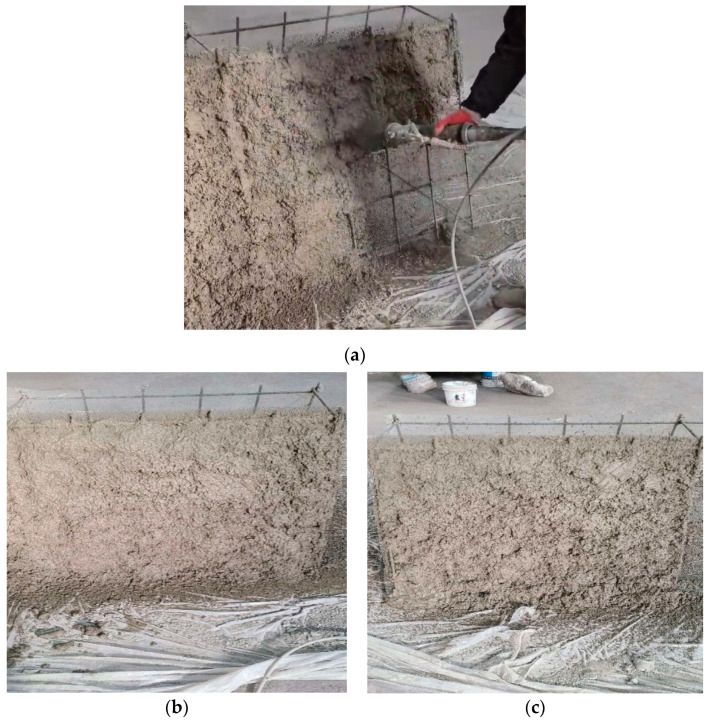
Shotcrete sprayed with different replacement ratios: (**a**) shotcrete spraying; (**b**) S-30; (**c**) S-50.

To evaluate the constructability of the mix, a shotcrete rebound test was conducted. The results indicate that only the mixtures with SiMn slag replacement ratios of 30% (S-30) and 50% (S-50) were feasible, with S-50 being identified as the most appropriate mix. The mixtures with SiMn slag replacement ratios of 70% (S-70) and 100% (S-100) showed the highest strength, but shotcrete construction application was not possible due to very low workability and the inability to be sprayed as shotcrete. It was observed that at the higher SiMn slag content, the mortar failed to pass through the hose during spraying, leading to backlogging. This can be attributed to the relatively heavier weight of the SiMn slag particles (e.g., a saturated surface-dry density of SiMn slag of 3 g/cm^3^), which is heavier than the commonly used silica sand (e.g., a saturated surface-dry density of 2.60 g/cm^3^). The rebound amounts for S-30 and S-50 mixes appeared visually similar, as shown in Figure 9. However, quantitative analysis revealed notable differences between the two mixtures of S-30 and S-50, demonstrating rebound rates of 51.2% and 18.5%, respectively. The higher rebound rate in S-30 was attributed to its relatively higher water content compared to the SiMn slag content, which resulted in inadequate adhesion of the mortar to the structure and a greater amount of material detachment during spraying. Consequently, the S-50 mix was determined to be the most suitable, as it exhibited lower material loss and ensured both physical and durability properties along with adequate workability.

**Figure 10:**
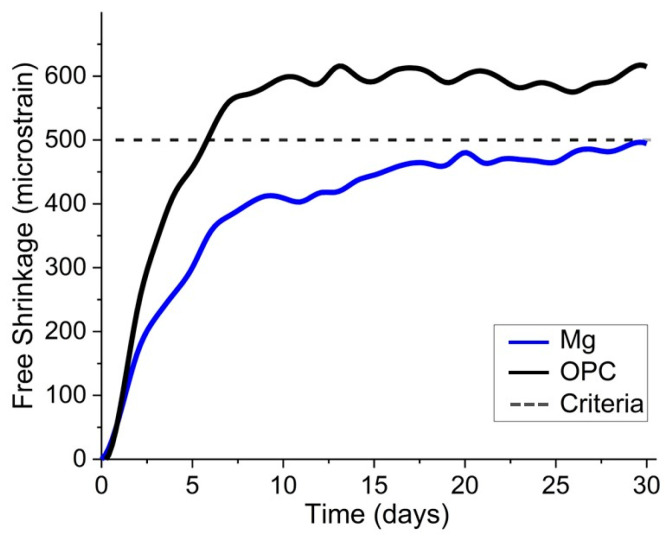
Free shrinkage strains of S-0 and S-50.

For serviceability and stability evaluation, a 30-day free drying shrinkage test was conducted. The shrinkage measurement results for S-0 and S-50 mixes are shown in Figure 10. The results demonstrated the significant effect of SiMn slag replacement on the shrinkage behavior. The S-0 mixture exhibited a free shrinkage strain of approximately 600 micro-strain, exceeding the criterion of 500 micro-strain, which is based on standard guidelines to prevent cracking and ensure durability. In contrast, the S-50 mixture stabilized at approximately 500 micro-strain, satisfying the criterion and demonstrating the effectiveness of replacing 50% of the fine aggregate with SiMn slag in reducing shrinkage strain. Both mixtures exhibited rapid shrinkage during the first 7 days, followed by gradual stabilization, indicating that most shrinkage occurs in the early curing phase, as expected. The results confirmed that the replacement of fine aggregate with SiMn slag enhances curing stability, likely due to the heavier density of the slag particles, which contributes to better internal restraint against shrinkage.

Based on the combined results of the rebound test and free drying shrinkage test, replacing 50% of the fine aggregate with SiMn slag is determined to be a suitable mixture for practical shotcrete construction applications where durability and constructability are critical. This replacement ratio not only ensures both mechanical and durability performance but also maintains sufficient constructability, making it an optimal mix.

## 4. Discussion

This study aimed to apply SiMn slag as a fine aggregate replacement in shotcrete mortar by adjusting replacement ratios and derived the optimal mix proportion suitable for the purpose. In addition, varying mix proportions using SiMn slag in shotcrete mortar were evaluated by assessing its influence on fresh properties, mechanical performance, durability, and constructability. To evaluate the practical applicability of SiMn slag, the study examined not only inherent material performances but also durability properties and constructability. The experimental findings confirmed that increasing SiMn slag content enhances mechanical strength and durability properties, making it a promising alternative material for sustainable construction applications. Among the evaluated durability indicators, enhanced resistance to freeze–thaw cycles and chloride ion penetration are especially important for shotcrete, which is primarily applied in structures exposed to external environments, such as landscape elements, tunnel linings, and bridge substructures. Such exposure demands high durability against cyclic freezing and chloride ingress to ensure long-term structural integrity.

Despite its beneficial effect on strength and durability, excessive SiMn slag replacement negatively impacted workability. Particularly, for the mixes with 70% and 100% replacement ratios, significant reductions in slump and spraying feasibility were observed. The rebound test results identified the S-50 mixture as the most suitable one, balancing mechanical performance with practical constructability while also exhibiting reduced shrinkage strain and improved adhesion during shotcrete application.

The findings of this study suggest that replacing 50% of fine aggregate with SiMn slag provides an optimal mix that ensures structural reliability while addressing sustainability concerns by reducing reliance on natural resources. However, this study does not provide a comprehensive theoretical assessment of potential chemical interactions between SiMn slag and cementitious components. Additionally, the risk of harmful substance leaching, especially under long-term exposure to moisture or aggressive environments, remains unexamined. These limitations should be addressed in future studies to ensure environmental safety and long-term durability.

Although the current production cost of SiMn slag mortar may exceed that of conventional natural fine aggregates, the overall life-cycle cost (LCC) could be comparable when considering environmental factors such as reduced carbon emissions, the conservation of natural resources, and the avoidance of environmental degradation. From a resource recycling perspective, the use of SiMn slag offers additional sustainability benefits, reinforcing its potential as a practical and eco-efficient alternative. Building upon this, future research should also aim to discover chemical interactions between SiMn slag and cementitious components, evaluate the economic viability of large-scale SiMn slag utilization, and validate its practical effectiveness through field implementation and long-term monitoring.

## 5. Conclusions

For industrial waste recycling purposes, an optimum mix proportion for shotcrete mortar was studied by varying replacement ratios (0%, 30%, 50%, 70%, and 100%) of SiMn slag as fine aggregate. Fresh and hardened performances of the mixture with various replacement ratios were evaluated to test the potential usage of SiMn slag as a sustainable construction material. The study results are as follows.

(1) The new mix proportions determined from preliminary tests have a water–cement ratio of 40% and a plasticizer content of 0.50% by weight of cement. Additionally, the unit weights of cement and water are 626 kg/m^3^ and 251 kg/m^3^, respectively. The total unit weight of aggregates including SiMn slag and fine aggregate is 1462 kg/m^3^.
(2) Fresh properties of the mortar were assessed by the air content and slump tests. As the SiMn slag replacement ratio increased incrementally from 0% to 100%, the average air content increased from 5.7% to 6.5% and the average slump decreased from 210 mm to 135 mm. The increase in air content and decrease in slump with higher SiMn slag replacement in fresh mortar performance suggest the need for mix adjustments to ensure practical applicability.
(3) Mechanical performances of the mortar were evaluated by the compressive, splitting tensile, and flexural strength tests. As the SiMn slag replacement ratio increased incrementally from 0% to 100%, the average compressive strength at 28 days increased from 24.9 MPa to 35.54 MPa. The splitting tensile and flexural strength at 28 days increased from 2.9 MPa to 4.1 MPa and from 6.25 MPa to 8.55 MPa, respectively. The results suggest that SiMn slag can be effectively utilized to enhance the structural capacity of mortar.
(4) Durability properties of the mortar were evaluated by the tests of chloride ion penetration, freeze–thaw resistance, and accelerated carbonation. As the SiMn slag replacement ratio increased incrementally from 0% to 100%, the chloride ion penetration resistance showed improvement as indicated by the test data of average charge decreasing from 4546 to 3326 coulombs for 28 days. From the freeze–thaw resistance test, the average of relative dynamic modulus of elasticity (RDME) after 300 cycles increased from 88.67% to 91.07%. From the carbonation test, the average carbonation depth decreased from 6.06 mm to 3.86 mm. The improved durability performance demonstrates the potential of SiMn slag for use in shotcrete structures exposed to aggressive environments.
(5) For the evaluation of constructability and serviceability of the mortar, the shotcrete rebound test and 30-day free drying shrinkage test were conducted. The rebound rates of the mortar with 30% and 50% replacement ratios were 51.2% and 18.5%, respectively, but the mortar with other replacement ratios was unsuitable for spraying. From the 30-day free drying shrinkage test, the mortar with a 50% replacement ratio exhibited below 500 micro-strain, satisfying the criterion of 500 micro-strain, whereas the mortar with a 0% replacement ratio exhibited approximately 600 micro-strain. The mortar with 50% SiMn slag replacement showed that SiMn slag can enhance both the efficiency of application and long-term performance in field conditions.
(6) The optimal shotcrete mix for sustainable construction was determined to be a mix consisting of 1462 kg/m^3^ of fine aggregate with 50% SiMn replacement (731 kg/m^3^), 626 kg/m^3^ of Ordinary Portland Cement (ASTM C150 Type I, equivalent to KS L 5201 Type 1), 251 kg/m^3^ of water, and 3.13 kg/m^3^ of polycarboxylate-based superplasticizer with air-entraining properties. The findings demonstrate the positive contribution of SiMn slag to the development and utilization of durable and sustainable recycled materials.

## Figures and Tables

**Table 1:** Waste process test.

Item	Unit	Detection Limit	Detection Criterion	Test Result
Pb	mg/L	0.040	3	0.086
Cu	mg/L	0.008	3	0.090
Cd	mg/L	0.002	0.3	0.008
Hg	mg/L	0.0005	0.005	-
Cr^6+^	mg/L	0.01	1.5	-
CN^−^	mg/L	0.01	1	-
As	mg/L	0.004	1.5	-
OPC	mg/L	0.0005	1	-
C_2_HCl_3_	mg/L	0.008	0.3	-
C_2_Cl_4_	mg/L	0.002	0.1	-
Cr	mg/L	0.01	-	-

**Table 2:** Physical properties of SiMn slag.

Property		Unit	Test Result
Density	Saturated Surface-Dry (SSD) Density	g/cm^3^	3.00
	Absolute Dry Density	g/cm^3^	2.97
Water Absorption		%	0.79

**Table 3:** Chemical composition of SiMn slag and fine aggregate.

Material	SiO_2_	Al_2_O_3_	Fe_2_O_3_	CaO	MgO	MnO	NaCl
Silicon Manganese Slag	37.1	12.1	0.73	23.5	4.69	17.1	0.007
Fine Aggregate	77.2	10.2	2.87	1.12	0.65	0.09	0.008

**Table 4:** Compressive strength results based on water–cement ratio conditions.

W/C (%)	Cement (kg/m^3^)	Water (kg/m^3^)	SiMn Slag (kg/m^3^)	Fine Aggregate (kg/m^3^)	Compressive Strength (MPa)		
					3 Days	7 Days	28 Days
30	626	188	1461	-	23.35	26.24	32.1
35	626	219	1461	-	27.8	30.7	36.8
40	626	251	1461	-	27.14	30.1	35.8
45	626	282	1461	-	25.2	28.2	33.1
50	626	313	1461	-	22.48	25.23	30.4

**Table 5:** Slump test results based on plasticizer conditions.

Plasticizer (C*%)	W/C (%)	Cement (kg/m^3^)	Water (kg/m^3^)	SiMn Slag (kg/m^3^)	Fine Aggregate (kg/m^3^)	Slump (mm)
0	40	626	251	1461	-	5
0.9	40	626	251	1461	-	FLOW
0.8	40	626	251	1461	-	27
0.7	40	626	251	1461	-	21
0.6	40	626	251	1461	-	16
0.5	40	626	251	1461	-	13
0.4	40	626	251	1461	-	11
0.3	40	626	251	1461	-	9

**Table 6:** Mortar mix proportion based on SiMn slag replacement rates.

Specimen	W/C (%)	Cement (kg/m^3^)	Water (kg/m^3^)	SiMn Slag (kg/m^3^)	Fine Aggregate (kg/m^3^)	Plasticizer (C*%)
S-0 (Control)	40	626	251	-	1462	0.50
S-30	40	626	251	439	1023	0.50
S-50	40	626	251	731	731	0.50
S-70	40	626	251	1023	439	0.50
S-100	40	626	251	1462	-	0.50

**Table 7:** Evaluation standard of total charge passage.

Total Passed Charge (Coulombs)	Chloride Ion Permeability
>4000	High
2000–4000	Moderate
1000–2000	Low
100–1000	Very low
<100	Negligible

**Table 8:** Average of air content test and slump test results.

Specimen	SiMn Slag (%)	Fine Aggregate (%)	Air Content (%)	Slump (mm)
Control	0	100	5.7	210
S-30	30	70	6.1	205
S-50	50	50	6	185
S-70	70	30	6.5	160
S-100	100	0	6.5	135

**Table 9:** Average of compressive strength test and splitting tensile strength test results.

Specimen	SiMn Slag (%)	Fine Aggregate (%)	Compressive Strength (MPa)				Splitting Tensile Strength (MPa)	
			3 Days	7 Days	14 Days	28 Days	14 Days	28 Days
Control	0	100	17.85	20.53	22.15	24.9	2.7	2.9
S-30	30	70	19.58	21.78	24.67	26.87	3.06	3.23
S-50	50	50	21.03	23.51	25.85	27.97	3	3.16
S-70	70	30	22.43	26.38	27.54	29.74	3.13	3.26
S-100	100	0	26.9	29.95	33.07	35.54	3.63	4.1

**Table 10:** Average of flexural strength test results.

Specimen	SiMn Slag (%)	Fine Aggregate (%)	Flexural Strength (MPa)	
			14 Days	28 Days
Control	0	100	5.55	6.25
S-30	30	70	6.3	7.15
S-50	50	50	6.75	7.4
S-70	70	30	7.4	7.65
S-100	100	0	8.2	8.55

**Table 11:** Average of chloride ion penetration, freezing and thawing, and accelerated carbonation test results.

Specimen	SiMn Slag (%)	Fine Aggregate (%)	Total Charge Passage (C)	Relative Dynamic Modulus of Elasticity (%)		Carbonation Depth (mm)
				0 Cycle	300 Cycles	
Control	0	100	4546	100	88.67	6.06
S-30	30	70	4086	100	90.36	4.45
S-50	50	50	4338	100	90	4.26
S-70	70	30	3667	100	90.77	3.93
S-100	100	0	3326	100	91.07	3.86

## Data Availability

The original contributions presented in the study are included in the article, further inquiries can be directed to the corresponding author.

## Abbreviations

The following abbreviations are used in this manuscript:

SiMn	Silicon Manganese
RDME	relative dynamic modulus of elasticity

## References

[1] Pierrehumbert R. (2019). There is no Plan B for dealing with the climate crisis. Bull. At. Sci..

[2] Bernardo G., Guida A., Mecca I. (2015). Advancements in Shotcrete Technology. Proceedings of the Structural Studies, Repairs and Maintenance of Heritage Architecture XIV.

[3] Ghiasi V., Omar H. (2011). Analysis of Shotcrete Lining of Underground Tunnels. Pertanika J. Sci. Technol..

[4] Lackner R., Macht J., Hellmich C., Mang H.A. (2002). Hybrid Method for Analysis of Segmented Shotcrete Tunnel Linings. J. Geotech. Geoenviron. Eng..

[5] Rispin M., Kleven O., Dimmock R., Myrdal R. Shotcrete: Early Strength and Re-Entry Revisited—Practices and Technology. Proceedings of the First International Conference on Underground Mining Technology.

[6] Moon T., Wang E., Oh J., Li K., Ramandi H.L., Zhang C., Saydam S. Evaluation of Shotcrete Performance from Full-Scale Load-Deflection Tests Using Numerical Analysis. Proceedings of the ARMA US Rock Mechanics/Geomechanics Symposium.

[7] Oreste P.P. (2003). A Procedure for Determining the Reaction Curve of Shotcrete Lining Considering Transient Conditions. Rock Mech. Rock Eng..

[8] Naseri S., Bahrani N. (2021). Design of Initial Shotcrete Lining for a Mine Shaft Using Two-Dimensional Finite Element Models Considering Excavation Advance Rate. Geotech. Geol. Eng..

[9] Bajwa D.S., Pourhashem G., Ullah A.H., Bajwa S.G. (2019). A concise review of current lignin production, applications, products and their environmental impact. Ind. Crop. Prod..

[10] Dong W., Kong F., He T., Liu M., Wu J., Zhang L. (2024). Enhancing performance and reducing carbon emissions in sprayed concrete using green silica-lignin admixture and recycled aggregates. Low-Carbon Mater. Green Constr..

[11] Garba M.J., Tian Y., Xie Z., Yu C., Hu C., Chen L., Yuan Q. (2024). Effect of accelerators on the long-term performance of shotcrete and its improvement strategies: A review. J. Build. Eng..

[12] Chen L., Huang L., Hua J., Chen Z., Wei L., Osman A.I., Fawzy S., Rooney D.W., Dong L., Yap P.-S. (2023). Green construction for low-carbon cities: A review. Environ. Chem. Lett..

[13] Mohanta N.R., Murmu M. (2022). Alternative coarse aggregate for sustainable and eco-friendly concrete—A review. J. Build. Eng..

[14] Tayeh B.A., Saffar D.M.A., Alyousef R. (2020). The Utilization of Recycled Aggregate in High Performance Concrete: A Review. J. Mater. Res. Technol..

[15] Akter S., Muniruzzaman M. (2021). Industrial Waste Management and Environment: A Study in Kamrangirchar (Raised Land), Dhaka. Environ. Manag. Sustain. Dev..

[16] Horii K., Tsutsumi N., Kitano Y., Kato T. (2013). Processing and reusing technologies for steelmaking slag. Nippon. Steel Tech. Rep..

[17] Ma Y., Moosavi-Khoonsari E., Kero I.T., Tranell G.M. (2018). Recycling of Industrial Byproducts in Construction: Metallurgical Slag as a Sustainable Aggregate. Metall. Mater. Trans. B.

[18] Olsen S.E., Tangstad M. Silicomanganese production—Process Understanding. Proceedings of the Infacon X.

[19] Choi H.B., Kim J.M. (2020). Properties of silicon manganese slag as an aggregate for concrete depending on cooling conditions. J. Mater. Cycles Waste Manag..

[20] Buruiana D.L., Obreja C.-D., Herbei E.E., Ghisman V. (2021). Re-Use of Silico-Manganese Slag. Sustainability.

[21] Ting J.J., Low W.W., Wong K.S., Ting T.Z.H., Abdul-Rahman H. (2023). Feasibility of recycling silicomanganese slag as a cementitious material. J. Eng. Technol. Adv..

[22] Makhambetov Y., Myngzhassar Y., Abdirashit A., Onuralp Y. (2024). Analysis of the silicomanganese market worldwide: A review. Eng. J. Satbayev Univ..

[23] Saha A.K., Khan M., Sarker P.K. (2018). Properties of Concrete Incorporating Recycled Materials. Resour. Conserv. Recycl..

[24] Wong N.H., Kong Z.Y., Sambo R., Chai C.S., Khoso A.R., Bamgbade J.A., Sunarso J. (2024). Physicochemical Characteristics of Silicomanganese Slag as a Recycling Construction Material: An Overview. Min. Metall. Explor..

[25] Patil A.V., Pande A.M. (2011). Strength Characteristics of Concrete Using Recycled Aggregates. Adv. Mater. Res..

[26] Nath S.K., Randhawa N.S., Kumar S. (2022). Environmental Impact of Concrete Incorporating Recycled Materials: A Comparative Study. Resour. Conserv. Recycl..

[27] (2024). Korean Industrial Standard: Standard Test Method for Density and Water Absorption Rate of Fine Aggregates.

[28] (2024). Korean Industrial Standard: Aggregates for Concrete.

[29] (2020). Korean Industrial Standard: Test Method for Compressive Strength of Concrete.

[30] (2021). Korean Industrial Standard: Portland Cement.

[31] (2016). Korean Industrial Standard: Standard Test Method for Air Content of Fresh Concrete by the Pressure Method.

[32] (2022). Korean Industrial Standard: Test Method for Concrete Slump.

[33] (2019). Korean Industrial Standard: Standard Test Method for Making Concrete Specimens.

[34] (2016). Korean Industrial Standard: Standard Test Method for Flexural Strength of Concrete.

[35] (2021). Korean Industrial Standard: Standard Test Method for Splitting Tensile Strength of Concrete.

[36] (2016). Korean Industrial Standard: Standard Test Method for Resistance of Concrete to Chloride Ion Penetration by Electrical Conductance.

[37] (2023). Korean Industrial Standard: Test Method for Resistance of Concrete to Rapid Freezing and Thawing.

[38] (2016). Korean Industrial Standard: Standard Test Method for Accelerated Carbonation of Concrete.

[39] (2021). Korean Industrial Standard: Standard Test Method for Drying Shrinkage Crack in Concrete.

